# Deep learning model for pleural effusion detection via active learning and pseudo-labeling: a multisite study

**DOI:** 10.1186/s12880-024-01260-1

**Published:** 2024-04-19

**Authors:** Joseph Chang, Bo-Ru Lin, Ti-Hao Wang, Chung-Ming Chen

**Affiliations:** 1 Department of Biomedical Engineering, College of Medicine and College of Engineering, National Taiwan University, No. 1, Sec. 1, Jen-Ai Road, Taipei 100, 100 Taipei, Taiwan; 2 The Data Science Degree Program, College of Electrical Engineering and Computer Science, National Taiwan University and Academia Sinica, Taipei, Taiwan; 3 Department of Radiation Oncology, China Medical University Hospital, Taichung, Taiwan; 4 Department of Medicine, China Medical University, Taichung, Taiwan; 5 EverFortune.AI Co., Ltd, Taichung, Taiwan

**Keywords:** Pleural effusion, Deep learning, Active learning, Chest radiographs, X-rays

## Abstract

**Background:**

The study aimed to develop and validate a deep learning-based Computer Aided Triage (CADt) algorithm for detecting pleural effusion in chest radiographs using an active learning (AL) framework. This is aimed at addressing the critical need for a clinical grade algorithm that can timely diagnose pleural effusion, which affects approximately 1.5 million people annually in the United States.

**Methods:**

In this multisite study, 10,599 chest radiographs from 2006 to 2018 were retrospectively collected from an institution in Taiwan to train the deep learning algorithm. The AL framework utilized significantly reduced the need for expert annotations. For external validation, the algorithm was tested on a multisite dataset of 600 chest radiographs from 22 clinical sites in the United States and Taiwan, which were annotated by three U.S. board-certified radiologists.

**Results:**

The CADt algorithm demonstrated high effectiveness in identifying pleural effusion, achieving a sensitivity of 0.95 (95% CI: [0.92, 0.97]) and a specificity of 0.97 (95% CI: [0.95, 0.99]). The area under the receiver operating characteristic curve (AUC) was 0.97 (95% DeLong’s CI: [0.95, 0.99]). Subgroup analyses showed that the algorithm maintained robust performance across various demographics and clinical settings.

**Conclusion:**

This study presents a novel approach in developing clinical grade CADt solutions for the diagnosis of pleural effusion. The AL-based CADt algorithm not only achieved high accuracy in detecting pleural effusion but also significantly reduced the workload required for clinical experts in annotating medical data. This method enhances the feasibility of employing advanced technological solutions for prompt and accurate diagnosis in medical settings.

## Introduction

Pleural effusion is the buildup of fluid in the pleural space and can be caused by a range of conditions such as congestive heart failure, cancer, pneumonia, and pulmonary embolism [1]. In the United States, pleural effusion affects around 1.5 million people each year [2]. Chest radiographs remain the primary imaging test for any patient with suspected pleural disease [3]. In many clinical scenarios, pleural effusion is life-threatening and timing of diagnosis and treatment is critical for patient outcome as it can be easily overlooked due to the high volume of images radiologists need to review [4].

To date, deep learning (DL) algorithms have been considered to provide promising results in detecting abnormalities in medical images [5–11]. However, most studies have used retrospectively annotated datasets, where the training and validation data are historically annotated and often derived from the same population distribution [12]. As a result, the findings may be biased and difficult to generalize to real-world clinical practice [13]. The reason behind this is due to a phenomenon called overfitting, where there is insufficient representative training data for DL algorithms to learn robust representations and draw generalizable conclusions on unseen data [14]. This issue is particularly common in medical imaging as it has been historically difficult to annotate large amounts of medical data due to cost and patient confidentiality [15].

Recent studies have found that DL algorithms can be trained to achieve optimal performance via an active learning (AL) framework [16]. This method iteratively goes through the training data and samples informative data points such that experts can focus on annotating more challenging cases. This approach would help significantly reduce the number of required expert annotations while allowing algorithms to train on a much larger dataset.

The aim of this study was to validate whether a DL-based Computer Aided Triage (CADt) algorithm can be developed under an active-learning framework to help reduce the workload for clinical experts while also producing robust performance for clinical practice across multisite data.

## Materials and methods

This retrospective study used data that were fully de-identified, anonymized and accessed under IRB CMUH106-REC3-118 with waived consent. This data was accessed on Feb 6th, 2023 for research purposes.

### Study design

**Fig. 1:**
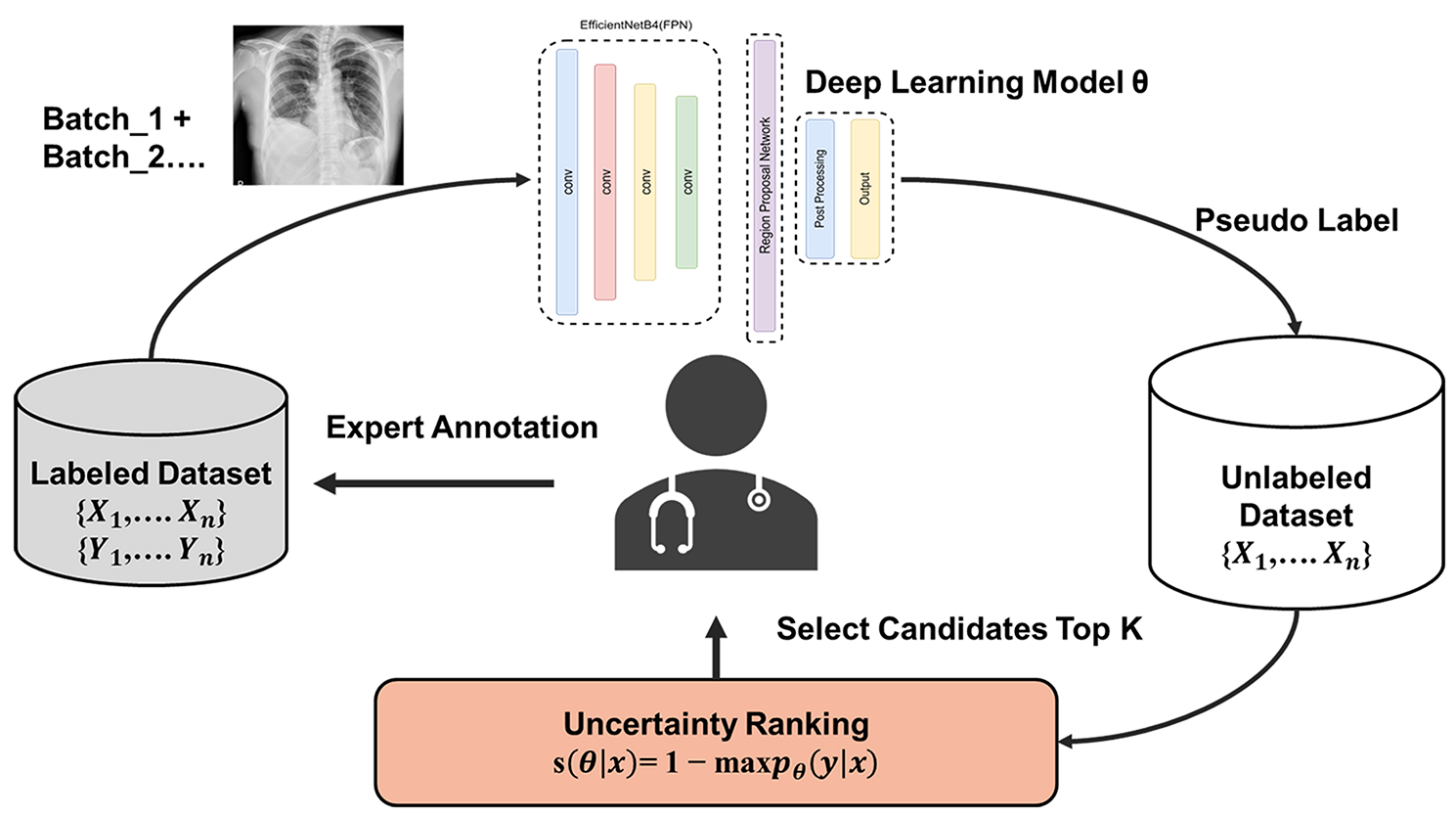
Active learning framework with pseudo labeling

The AI algorithm was trained using a development data set of 10,599 anonymized chest radiographs and consecutively collected from an institution in Taiwan between 2006 and 2018. This data set was stratified based on whether pleural effusion is present and randomly split into training (80%), validation (10%), and testing (10%). The testing set was used for internal validation. This data set was annotated by expert radiologists in Taiwan. A deep learning algorithm was trained based on the “Detectron2” [17] framework where data augmentations such as random rotation, flipping, translation, resizing was performed during training. A threshold of 0.5 was set as the cut off value for the probability score in indicating whether pleural effusion was present in each radiograph and the pipeline was further revised such that the algorithm could be trained via active learning where the parameters were updated using the stochastic gradient descent with a batch size of four. The final output produces a binary result tailored for clinical triage purposes. Initially, the algorithm was trained using the development data set from Taiwan and a randomly pooled (10%) set of the training data was used for initial expert annotation to develop the first baseline algorithm [18]. The objective of the AL framework was to continually update the algorithm by iteratively going through the data and sampling the most informative examples for annotation to minimize annotation efforts. There are many sampling methods in active learning. The approach conducted in this study was to use the uncertainty method [19–21]. This methodology employs a sampling technique that selects batches of data with high uncertainty scores for expert annotation in each iteration. Uncertainty is assessed using the Difficulty Calibrated Uncertainty Sampling (DCUS) method, inspired by [22]. This method combines category-wise entropy and object detection entropy to calculate the “difficulty” of samples, which then informs the AL pooling criteria. Furthermore, in each iteration, the algorithm selects highly confident data with low uncertainty scores to serve as pseudo-labeled data. These are then incorporated into the training set for the subsequent iteration, effectively combining active learning with pseudo labeling techniques. The number of iterations used was 212,000 determined based on the hyperparameter optimization result of a batch size of four and updated the parameters using a stochastic gradient algorithm. Studies have shown that expert annotations can be reduced to 90% using the active learning framework, and thus for each iteration, predicted data with uncertain scores are selected for expert annotation [23]. The remaining 90% of the training data utilized a semi-supervised method by using the pseudo labels generated by the algorithm and treated them as ground truth during training. Many studies have shown that pseudo labels provide consistency regularization and improved performance as the algorithm goes through all of the training data with minimal annotated data [24]. The overall training design is summarized in Fig. 1.

An external validation was then designed to further validate the algorithm’s performance against expert radiologists from the United States (U.S.) through a retrospective standalone performance study with multi-readers and multisite data sets from the U.S. and Taiwan.

The code underlying this work can be found online at https://github.com/facebookresearch/detectron2.

### External validation data collection

**Fig. 2:**
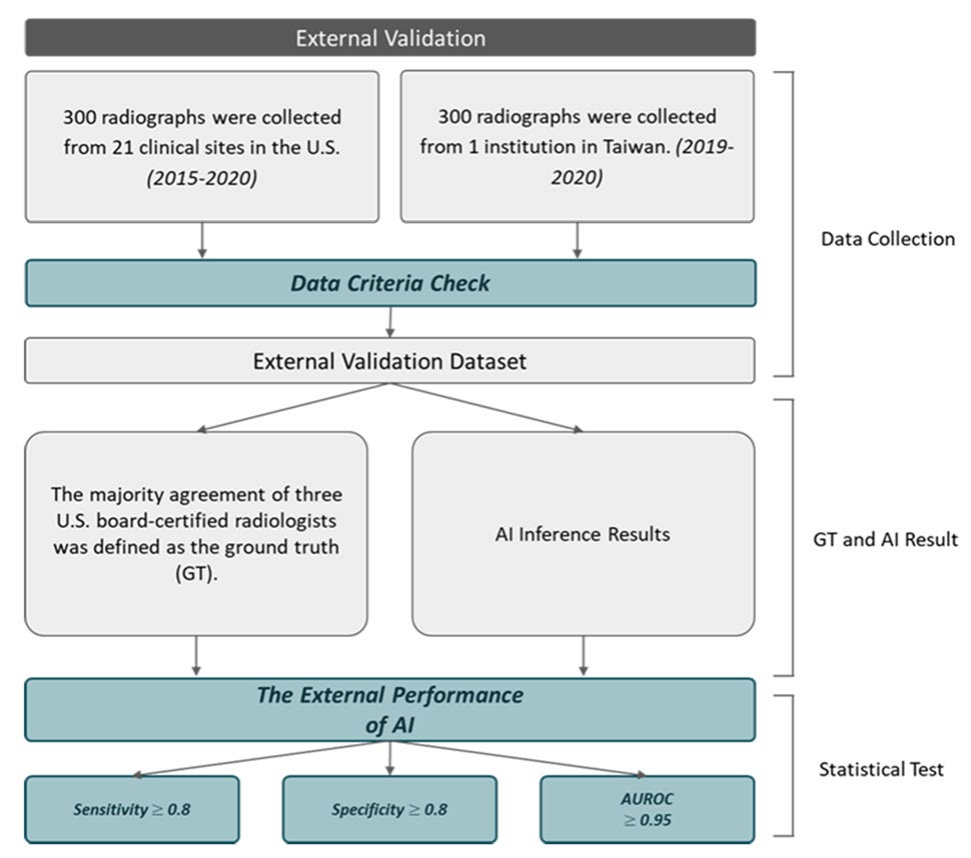
Flowchart of the validation workflow

A total of 600 anonymized chest radiographs were consecutively collected between 2015 and 2020 from 21 clinical sites in the U.S. and between 2019 and 2020 from 1 clinical site in Taiwan. The data set were generated from 13 different manufacturers of radiologic data source: Samsung Electronics, Shimadzu, Toshiba, Onica Minolta, GE Healthcare, Drtech, Canon Inc., Siemens, Oehm und Rehbein GmbH, Philips Medical Systems, Swissray, Kodak, Agfa, Fujifilm. The radiographs were collected following the inclusion and exclusion criteria with stratification of whether the patient received thoracentesis as shown in Fig. 2.

The global criteria for the intended use patient population in this study are defined as: greater or equal to 18 years old in both females and males. For the chest x-ray image, the images were enrolled by using the following inclusion criteria: (1) the chin should not be superimposing any structures, (2) arms are not superimposed over the lateral chest wall (this can mimic pleural thickening), (3) minimal superimposition of the scapulae borders on the lung fields is acceptable, (4) sternoclavicular joints are equidistant from the spinous process, (5) the clavicle is in the same horizontal plane, (6) a maximum of ten posterior ribs are visualized above the diaphragm, (7) the 5th -7th anterior ribs should intersect the diaphragm at midclavicular line, (8) the ribs and thoracic cage are seen only faintly over the heart, and (9) clear vascular markings of the lungs should be visible.

### Ground truth definition

The ground truthing of this assessment study included three U.S. board-certified expert radiologists reviewing the radiographs, and assigning whether a pleural effusion is present in each image. These radiologists all have the U.S. American Board of Radiology (ABR) board-certified in Diagnostic Radiology with greater than 10 years of experience in assessing Chest X-Ray and conducted a high volume (greater than 75 cases per week) of CXR assessments.

For each case, each radiologist was asked to provide the following information: pleural effusion is present or absent, size (small/moderate/large) [25] and location (right/left/bilateral) of pleural effusion, and any additional comments the radiologist would like to provide about the case. It is worth noting, despite the common use of qualitative terms such as small, moderate, and large to describe pleural effusion sizes and factors such as blunting of the costophrenic angle, partial filling of the pleural space and substantial opacification of the hemithorax, there currently isn’t a standardized grading system universally accepted in the clinical community [25]. Consequently, the categorization of effusion size in this study is inherently based on the individual clinical judgment of each reviewing radiologist. The presence, size, and location of pleural effusion were determined based on the majority agreement of three U.S. board-certified expert radiologists who reviewed the radiographs independently and was further defined as the ground truth (GT).

### Statistical analysis

Data processing and statistical analyses were conducted using Python 3.6 and R 4.0.2. A chi-square test was used to test for independence between the categorical variables determining whether there was a statistically significant association between the variables or whether they occur independently of each other. One-Sample Z tests were adopted as the testing method to verify the sensitivity, specificity, and AUROC (Area Under the Receiver Operating Characteristic curve) of the AI algorithm respectively, against the GT. The 95% confidence intervals (95% C.I.) of the sensitivity, specificity and the AUROC of the AI algorithm were calculated to evaluate the performance of detecting pleural effusion. Notably, we employed DeLong’s method for computing the 95% CIs of the AUROC, which is particularly suited for correlated ROC curves, providing a more accurate assessment of the algorithm’s diagnostic performance. This method adjusts for the correlation between the AUROC estimates, offering a rigorous statistical foundation for evaluating model accuracy. Additionally, Wilson’s score method was used for calculating the 95% CIs for sensitivity and specificity. This approach is advantageous for binomial proportions, particularly in situations where the sample size is small or the event rate is very low or high, as it produces intervals that are more accurate and closer to the true population parameter than those obtained using simpler approximations.

An upper-tail test was used in the study where the significant level (Type I error, α) was 2.5%. This statistical framework supported our efforts to ensure the AI algorithm’s generalizability across different subpopulations, including variations by gender (male/female), data source (U.S./Taiwan), and manufacturer (Samsung Electronics, Shimadzu, Toshiba, etc.). Analyses also extended to sensitivity for pleural effusion size (small/moderate/large) and location (right/left/bilateral), and an examination of potential confounders such as image quality issues or radiologic findings unrelated to pleural effusion, to determine their systematic impact on the AI’s performance.

## Results

### Patient characteristics

A total of 600 chest X-ray PA view images that met the inclusion criteria were consecutively selected for the study. Among them, 332 (55.3%) were male and 266 (44.3%) were female. The mean (standard deviation, SD) age was 58.7 (17.7) years. The case distribution was performed as detailed in Table 1 across the presence of pleural effusion or not.

**Table 1:** Basic characteristics for 600 external validation dataset

	Cases (N)	Pleural Effusion ^a^		*P*-value
		Presence	Absence	
Gender				0.0759
Female	266	116	150	
Male	332	169	163	
N/A^**b**^	2	1	1	
Age Group				< 0.0001
18–49 y/o	171	30	141	
50–64 y/o	186	103	83	
Above 65 y/o	242	153	89	
N/A^b^	1	0	1	
Data Source				< 0.0001
US	300	160	140	
Taiwan	300	126	174	
Manufacturer				0.0004
Samsung Electronics	135	64	71	
Shimadzu	159	67	92	
Toshiba	152	60	92	
Others^**c**^	154	95	59	
Size of Pleural Effusion				-
Small	196	196	-	
Moderate	73	73	-	
Large	16	16	-	
Size undefined^**d**^	1	1	-	
Location of Pleural Effusion				-
Right	134	134	-	
Left	85	85	-	
Bilateral	62	62	-	
Location undefined^**d**^	5	5	-	

^a^ Presence and absence of pleural effusion cases were defined based on the majority agreement between the three radiologists.

^b^ Cases’ where gender and age were unknown in the dataset.

^c^ Other X-ray manufacturers include Konica Minolta, GE Healthcare, Drtech, Canon Inc., Siemens, Oehm und Rehbein GmbH, Philips Medical Systems, Swissray, Kodak, Agfa, Fujifilm, and unknown.

^d^ Cases where only two radiologists agreed on the presence of pleural effusion and the size or location of the pleural effusion was in disagreement between the two radiologists. Thus, these cases were marked as undefine.

### Radiologist consistency analysis

Out of the 600 cases, we collected 1,800 pleural effusion assessments from three (3) radiologists. The consistency between the three (3) radiologists was evaluated using Cohen’s kappa to assess the agreement between each pair of radiologists in assessing the pleural effusion in 600 cases. According to the strength of the agreement based on Cohen’s Kappa value, they all showed high agreement between any two of the radiologists, kappa = 0.84 (95% confidence interval [CI], 0.80 to 0.89), 0.8 (95% CI, 0.80 to 0.89) and 0.89 (95% CI, 0.85 to 0.92), respectively, with all *p* < 0.0001 (Table 2).

**Table 2:** Truthers consistency

	Kappa value	95% CI	*p*-value
Radiologists1 vs. Radiologists2	0.84	(0.80, 0.89)	< 0.0001
Radiologists1 vs. Radiologists3	0.84	(0.80, 0.89)	< 0.0001
Radiologists2 vs. Radiologists3	0.89	(0.85, 0.92)	< 0.0001

#### Evaluation of standalone AI performance

The primary outcomes were the sensitivity, specificity and AUC per case. For the model performance to be acceptable for future clinical use, the performance goals were set in accordance to the US Food and Drug Administration (FDA) regulatory guidelines [24] requiring such CADt devices to at least reach a sensitivity/specificity of over 0.8 and an AUC of greater or equal to 0.95 [26]. As shown in Table 3, the One-Sample Z tests showed the sensitivity and specificity both exceed 0.8, as well as an AUC exceed 0.95. The sensitivity of the AI algorithm was 0.95 with a 95% CI of [0.92, 0.97], the specificity was 0.97 with a 95% CI of [0.95, 0.99], and the AUC was 0.97 with a 95% DeLong’s CI of [0.95, 0.99]. Overall, the agreement between the AI algorithm and GT met the performance goal of exceeding 0.8 in both sensitivity and specificity, and AUC exceeding 0.95, compared with the GT.

**Table 3:** Performance of the AI algorithm by each subpopulation

	Sensitivity (95% Wilson CI)	Specificity (95% Wilson CI)	AUC (95% DeLong’s CI)
Overall	0.95 (0.92, 0.97)	0.97 (0.95, 0.99)	0.97 (0.95, 0.99)
**Gender**			
Female	0.93 (0.87, 0.96)	0.98 (0.94, 0.99)	0.96 (0.92, 0.99)
Male	0.96 (0.92, 0.98)	0.97 (0.93, 0.99)	0.98 (0.96, 1.00)
**Age Group**			
18–49 y/o	0.93 (0.79, 0.98)	0.99 (0.95, 1.00)	0.96 (0.90, 1.00)
50–64 y/o	0.99 (0.95, 1.00)	0.96 (0.90, 0.99)	0.98 (0.95, 1.00)
Above 65 y/o	0.93 (0.88, 0.96)	0.97 (0.91, 0.99)	0.96 (0.94, 0.99)
**Data Source**			
US	0.92 (0.87, 0.95)	0.96 (0.91, 0.98)	0.95 (0.92, 0.98)
Taiwan	0.99 (0.96, 1.00)	0.99 (0.96, 1.00)	1.00 (0.99, 1.00)
**Manufacturer**			
Samsung Electronics	0.97 (0.89, 0.99)	0.99 (0.92, 1.00)	1.00 (0.99, 1.00)
Shimadzu	1.00 (0.95, 1.00)	0.99 (0.94, 1.00)	0.99 (0.98, 1.00)
Toshiba	0.98 (0.91, 1.00)	0.99 (0.94, 1.00)	1.00 (1.00, 1.00)
Others^a^	0.88 (0.80, 0.93)	0.92 (0.82, 0.96)	0.91 (0.86, 0.96)
**Size of Pleural Effusion**			
Small	0.96 (0.93, 0.98)	-	
Moderate	0.90 (0.82, 0.95)	-	
Large	1.00 (0.81, 1.00)	-	
**Location of Pleural Effusion**			
Right	0.92 (0.86, 0.95)	-	
Left	0.99 (0.94, 1.00)	-	
Bilateral	0.97 (0.89, 0.99)	-	

^a^ Other X-ray manufacturers include Konica Minolta, GE Healthcare, Drtech, Canon Inc., Siemens, Oehm und Rehbein GmbH, Philips Medical Systems, Swissray, Kodak, Agfa, Fujifilm, and unknown.

**Fig. 3:**
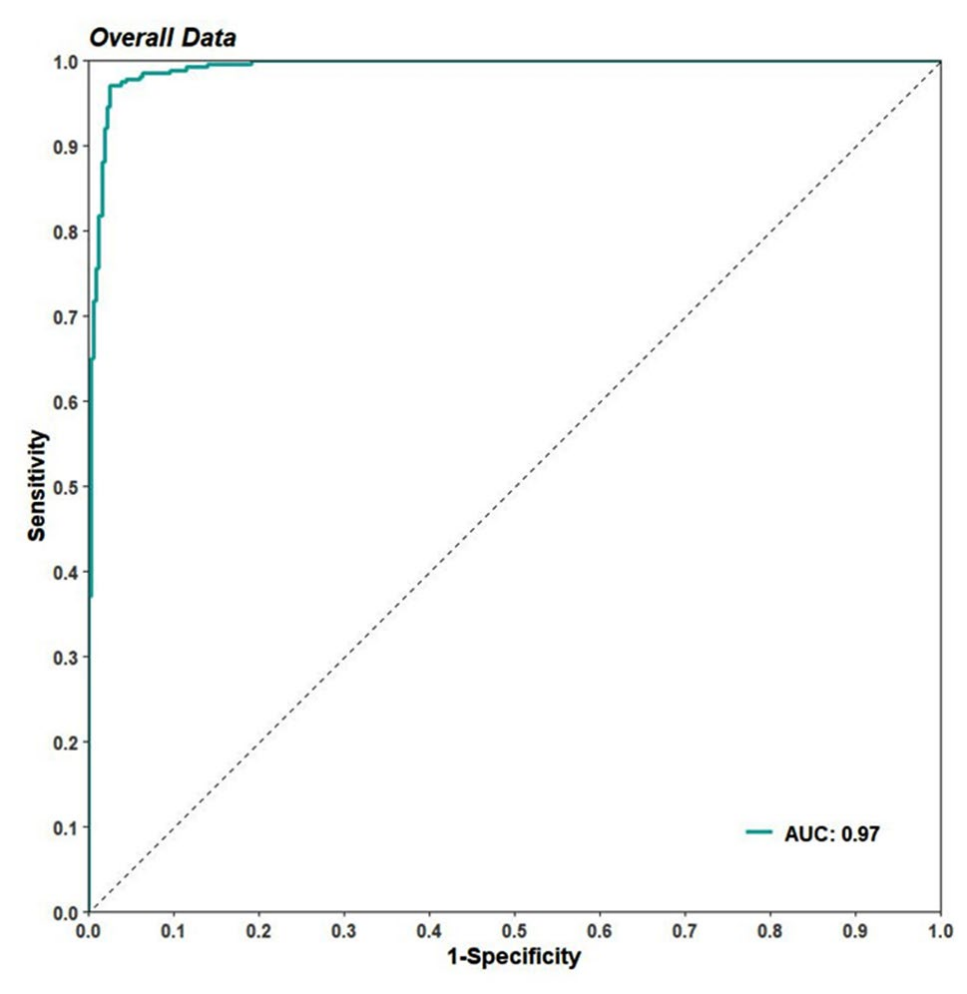
The ROC curve of the AI algorithm against the GT (empirical)

The ROC curve (Fig. 3) provides a visualized depiction of the AI algorithm’s performance, we can see the AUC measures the entire two-dimensional area underneath the empirical ROC curve at all classification thresholds from (0,0) to (1,1) was 0.97. For the 600 cases, the AUC of the AI algorithm indicates the model is able to demonstrate high classification performance.

### Subgroup Analysis results

Subgroup analysis across different subpopulations was also performed assessing the generalizability of the AI algorithm’s performance (Table 3). By assessing the AI system’s performance against ground truth (GT) for specific subgroups ensures that the system is reliable and effective across a diverse range of patient populations.

The study results indicate that all subpopulations performed well in accurately detecting pleural effusion with sensitivity ranging from 0.90 to 1.00 and specificity ranging from 0.92 to 0.99. These findings demonstrate the effectiveness of the AI algorithm in assessing pleural effusion in diverse patient populations, regardless of their demographic or clinical characteristics.

**Fig. 4:**
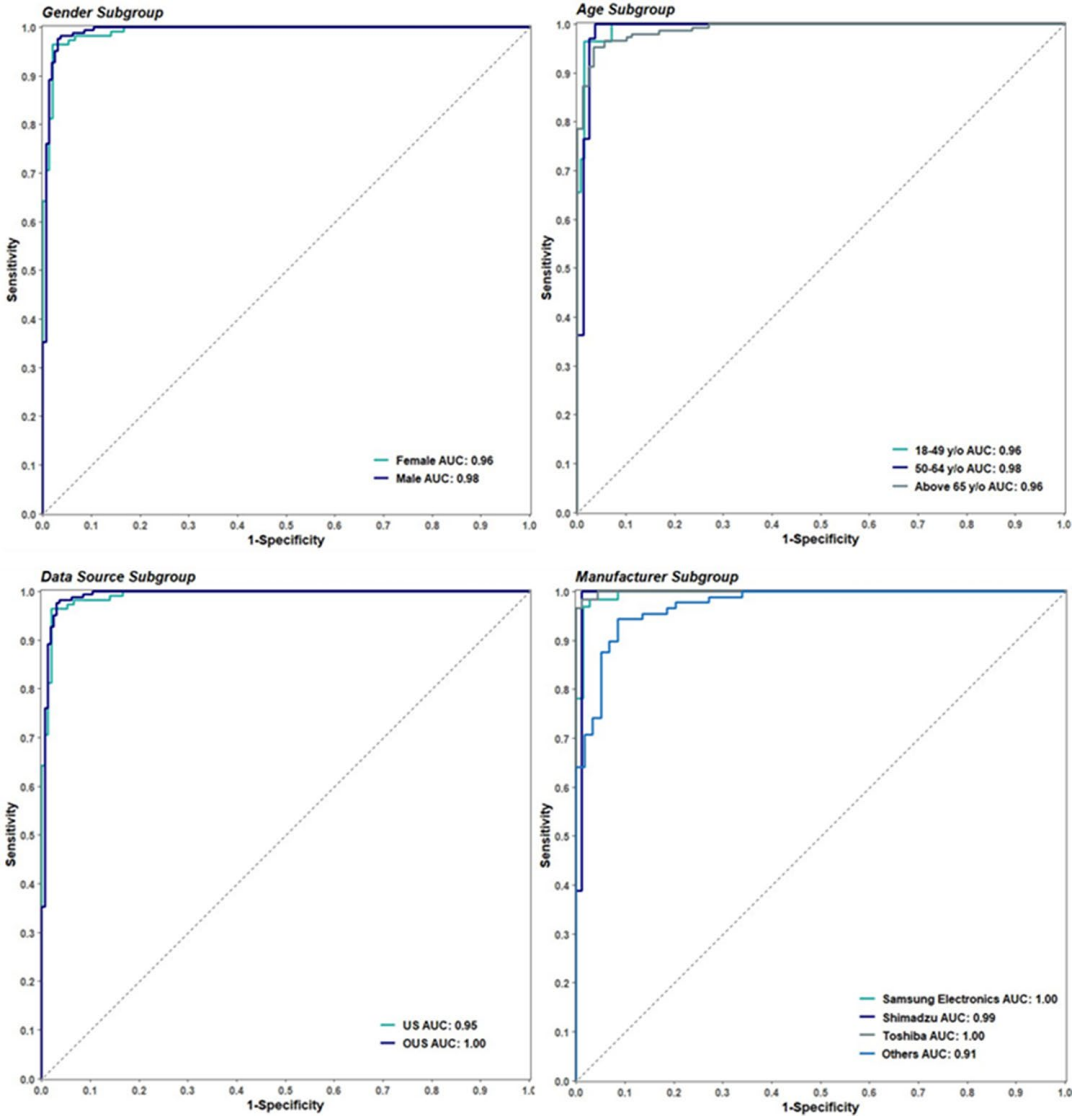
The ROC curve of the AI algorithm across all subgroups

Figure 4 displays how each subpopulation corresponds to an empirical ROC curve, indicating the device’s consistent performance across all subgroups.

### Confounding analysis results

Besides the subgroup analysis, radiologists reported 25 cases accompanied radiologic findings other than pleural effusion and 7 cases with image quality issues presented in the radiograph. A single case may exist with one or more radiologic findings and/or image quality-related issues. As shown in Table 4, the AI algorithm’s performance was evaluated by comparing it with GT, and cases accompanied by radiologic findings showed 15 TP, 9 TN, 0 FN, and 1 FP. For cases with image quality issues, the results were 3 TP, 3 TN, 1 FN, and 0 FP. These results demonstrate that the AI algorithm can perform effectively even when faced with possible confounding factors.

**Table 4:** Performance of the AI algorithm by Each Subpopulation

	Sensitivity (95% Wilson CI)	Specificity (95% Wilson CI)	AUROC (95% DeLong’s CI)
Radiologic Findings^**a**^	1.00 (0.80, 1.00)	0.90 (0.60, 0.99)	0.97 (0.90, 1.00)
Image Quality Issues^**b**^	0.75 (0.30, 0.99)	1.00 (0.44, 1.00)	0.92 (0.69, 1.00)

^a^ Cases with radiologic findings include possible confounders as Air-fluid Level, Airspace Disease, Atelectasis, Blebs, Cardiomegaly, Fracture, Infiltrate, Mass, Nodule, Obstructive Airways Disease, Pleural Effusion, Pneumonia, and Scoliosis

^b^ Cases with image quality issues include possible confounders as Anatomy not complete, Artifact present, Field of view issues, and Others

### Active learning results

**Fig. 5:**
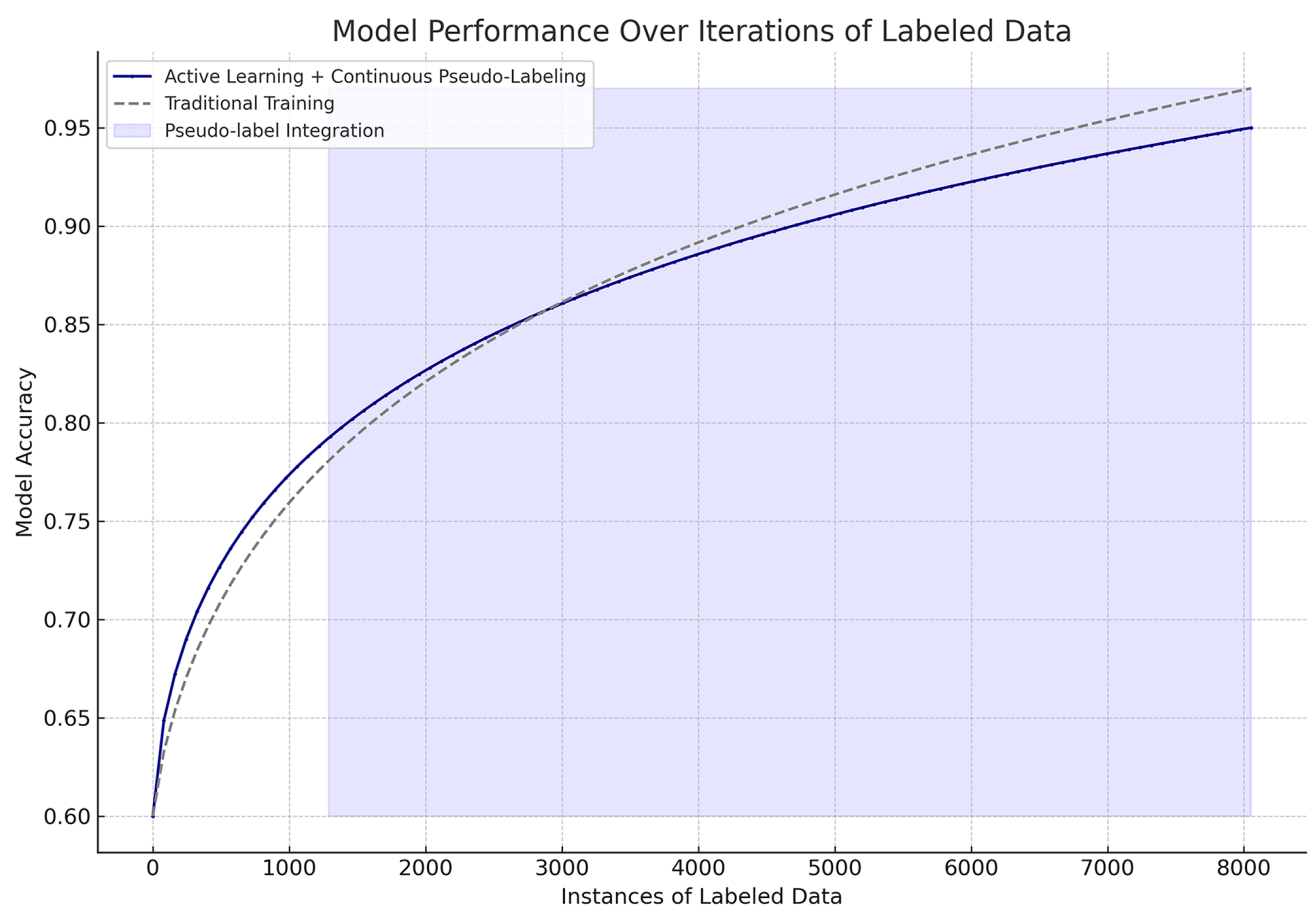
Model performance over labeled data via AL vs. traditional learning

As shown in Table 5, we observe an aggregate of 8,053 chest radiographs available for the training set. With the implementation of the AL framework, an iterative processing of each data subset revealed the necessity for expert annotation in a collection of 549 radiographs, which constituted 38.1% of the positive cases. The results also indicate that the AL framework pooled a higher percentage of small pleural effusion radiographs within the positive cohort for expert annotations with 23.2% of the pleural effusion positive radiographs being small. Conversely, the algorithm identified 740 radiographs within the negative cohort that needed expert annotation, representing 11.1% of the total negative cases. Overall, a total of 1,289 radiographs, 16% of the total training set were required for expert annotation. Figure 5 presents the performance comparison between the AL framework with continuous pseudo-labeling and traditional training methods over the utilization of labeled data. The AL approach, supplemented by iterative incorporation of pseudo-labeled data, achieved a model accuracy of 95%. This was accomplished by using 1,289 manually annotated radiographs by radiologists and 6,764 pseudo-labeled radiographs. In contrast, the traditional training method, utilizing the entire set of 8,053 labeled samples by radiologists, reached a higher model accuracy of 97%. The AL strategy demonstrated a gradual increase in accuracy in the initial phases, indicative of the strategic selection of challenging cases for manual annotation. This was followed by the inclusion of high-confidence pseudo-labeled instances, which contributed to refining model performance. The traditional training model’s accuracy increased more steeply, plateauing at the final accuracy percentage upon the inclusion of the complete labeled dataset. These results indicate that the AL framework, through the use of pseudo-labeling, can achieve near-comparable performance to traditional training methods with fewer manually labeled instances. The AL model’s progression reflects an effective use of expert annotations, achieving significant accuracy levels with a combined strategy of manual and pseudo-labeling.

**Table 5:** Ground truth data (training set) via active learning (AL)

Dataset	Available ^a^	With AL ^b^	Percentage
Pleural Effusion Positive	1438	549	38.1%
Small		334	23.2%
Moderate		113	7.9%
Large		102	7.0%
Pleural Effusion Negative	6615	740	11.1%
Total	8053	1289	16.0%

^a^ Total number of available cases

^b^ Total number of labeled cases that was used via active learning

### Comparison of CADt algorithms

In this section, as shown in Table 6, we show the comparative analysis of our proposed CADt algorithm against existing US FDA-approved AI algorithms for the detection of pleural effusion. The benchmarks for performance metrics include sensitivity, specificity, and AUC. As mentioned above, the primary outcomes are to reach a sensitivity and specificity of at least 0.8 and an AUC of 0.95. Our proposed algorithm reached a sensitivity of 95%, specificity of 97%, and an AUC of 0.97 meeting performance standards against these existing leading FDA-approved AI solutions for pleural effusion detection.

**Table 6:** CADt Performance of FDA Approved AI Algorithms [24]

Device	Intended Use	Modality	Sensitivity/Speficity	AUC
HealthCXR	Pleural Effusion	Chest X-ray	96/93	0.98
qXR-PTX-PE	Pleural Effusion	Chest X-ray	96/94	0.98
Lunit CXR Triage	Pleural Effusion	Chest X-ray	88/90	0.96
Our proposed algorithm	Pleural Effusion	Chest X-ray	95/97	0.97

## Discussion

In this study, we introduced a novel AL framework to build a clinical grade AI algorithm aimed at detecting pleural effusion in chest radiographs with high performance and minimal expert annotation effort. Unlike direct comparisons with previous studies, which may involve different datasets and tasks, our emphasis is on the methodological advancements and the clinical relevance of our approach. Previous works, such as those by Singh et al. and Ajmera et al., have contributed valuable insights into the application of AI in radiology [27–28]; however, our approach distinguishes itself through the use of an AL and semi-supervised learning strategy that reduces the need for expert-labeled data. This novel approach represents a step forward in developing clinical-grade AI tools with reduced resource intensity. We further used an external multisite data set from the U.S. and Taiwan that included multiple different types of manufacturers to demonstrate the robust generalization capacity of the algorithm.

Our algorithm can interpret full-size high-spatial-resolution chest radiographs sent directly from any picture archiving and communication systems (PACS) used in the daily clinical practice. The observed results of the standalone performance validation study demonstrated that the AI algorithm by itself, in the absence of any interaction with a clinician, can assess pleural effusion in chest radiographs with high consistency with U.S. expert radiologists. The algorithm demonstrated a high sensitivity and specificity of 0.95 with a 95% CI of [0.92, 0.97] and the specificity is 0.97 with a 95% CI of [0.95, 0.99], respectively. The algorithm also showed an AUC performance of 0.97 with a 95% DeLong’s CI of [0.95, 0.99]. These results show comparable performance against other US FDA-approved market ready pleural effusion detection software as shown in Table 6]. It is also worth noting that our framework was able to produce robust performance across different sizes of pleural effusion ranging from small to large with an AUC of 0.96, 0.90 and 1.0 respectively. In comparison against previous studies that have only shown single level performances [29–30], our current study has shown extensive subgroup analysis to demonstrate robust performance across different severity levels (small, medium, large), location (right, left, bilateral), gender, age groups and manufacturers. One of the main limitations of previous studies is the lack of performance analysis on potential confounding factors and subgroups. This is particularly important as this will often affect the AI’s performance if the algorithm is not robust enough. The performance across different subpopulations needs to be of high sensitivity and specificity across each subgroup to be clinically relevant. Other potential confounding factors as shown in Table 4, such as mass, atelectasis, airspace disease, air-fluid level, fracture, pseudotumor, infiltrate, pneumonia, blebs, miliary disease, postoperative change, pulmonary fibrosis were also considered and tested and showed that they do not systematically affect the algorithm’s performance.

**Fig. 6:**
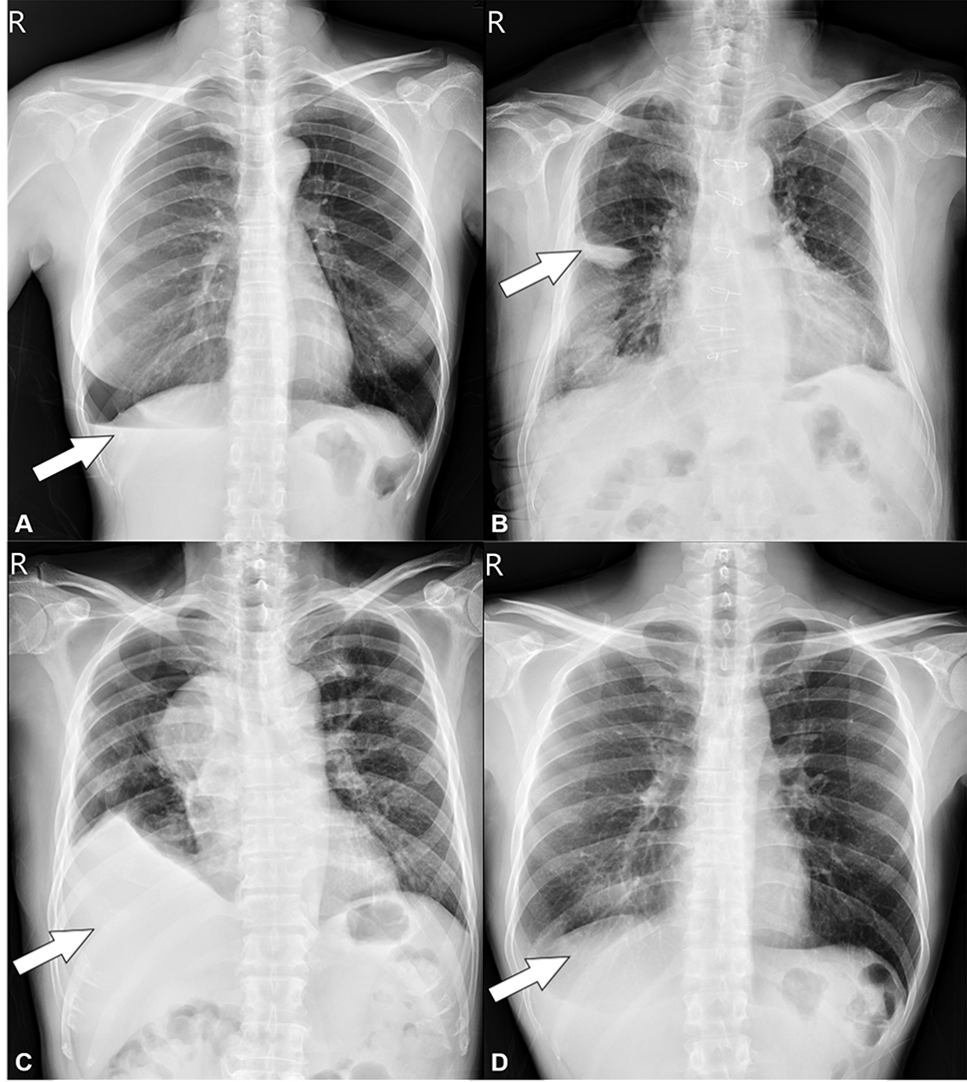
Challenging cases selected by the algorithm during training (**A**) right pneumothorax with associated pleural effusion and visible air-fluid level, (**B**) right lung laceration with associated pleural effusion, (**C**) pneumothorax, mediastinal mass with pleural effusion, and elevated diaphragm, (**D**) right-sided blunting of the costophrenic angle with minimal pleural effusion

The application of combining active learning and semi-supervised training via psuedo-labeling in this study demonstrated its potential to reduce the expert annotation efforts required for developing clinical-grade AI algorithms. By implementing an AL framework experts were allowed to focus on reviewing only challenging cases selected by the algorithm (16%) while the algorithm utilizes pseudo-labels for the remaining training data. The strategic utilization of a subset of the dataset for expert labeling, supplemented by pseudo-labeled data for model training, suggests potential towards more efficient AI algorithm development. In addition to reducing annotation efforts, our active learning approach was able to identify clinically challenging cases using the uncertainty method during the training process. As shown in Fig. 6 below, several challenging cases such as coexisting air and fluid, obscured fluid accumulation due to lung tissue injury, and minimal pleural effusion with subtle changes in the costophrenic angle were detected as selected candidates for confirmation and annotation. These finding suggests that active learning can help focus radiologist’s attention on challenging cases that may require additional clinical scrutiny. Our study provides evidence that active learning is an effective strategy for identifying challenging cases that can be particularly useful in clinical practice.

One of the main limitations of our study was it was retrospective in nature and thus all radiographs were de-identified without any relevant clinical information or patient history for experts to consider. In a real clinical setting, clinicians would be able to examine the patient and obtain detailed history to identify the area of concern before or during looking at the radiographs, thus improving meaningful clinical interpretation.

In conclusion, this study introduces a novel framework for developing CADt tools via AL and semi-supervised learning, highlighting a reduction in the need for extensive expert radiologist annotation while ensuring performance that is on par with existing FDA-approved solutions. Instead of drawing direct comparisons with previous methods based on different datasets and tasks, our focus has been on the methodological advancements and the practical benefits these bring to clinical settings. The clinical implications of our approach extend beyond achieving high-performance metrics. By demonstrating that a high-quality algorithm can be developed with less expert annotation, we present a method that not only optimizes the use of radiological expertise by concentrating on more challenging cases identified through the AL process but also significantly reduces the resources and time typically required for training clinical-grade AI tools. By prioritizing the reduction of expert burden and demonstrating a path to maintain diagnostic accuracy, our approach offers an alternative approach in developing practical clinical grade AI algorithms.

## Data Availability

The datasets analyzed in the current study are not publicly available due to ethical restrictions and the proprietary nature of the study. Requests for access to the data can be directed to JC, the first author of this manuscript. For further information or inquiries regarding the data, please contact JC at jochang920@gmail.com.
